# Supplementary material for: Causal Inference for First Non‐Fatal Events With the Competing Risk of Death: A Principal Stratification Approach
Source: Stat Med. 2025 Nov 18;44(25-27):e70311. doi: 10.1002/sim.70311 (PMC12625808; doi:10.1002/sim.70311)
Supplement: Supplementary file 1 — Data files: sim70311‐sup‐0001‐DataFiles.zip. [file SIM-44-0-s001.zip › codes/README.docx]

**functions.R:** Define a list of functions for future use.

**simulation_ig.R:** Simulation studies with the inverse-Gaussian distributed baseline frailty

**simulation_gamma.R:** Simulation studies with the gamma distributed baseline frailty

**example_est.R:** Application to the COPERNICUS trial. The point estimates are obtained.

**example_est_data.R:** Application to the COPERNICUS trial. To generate the “byproduct” data from which the point estimates are obtained. The “byproduct” data will be used in the proportionality test.

**example_CI.R:** Application to the COPERNICUS trial. The bootstrap confidence intervals are obtained.

**(The COPERNICUS Trial Data is not Publicly Sharable)**

**proportionality.R:** The proportionality test for the principal stratum hazards model

Because the codes were run on the server, they require a text file containing the input parameters. The following R codes generate the parameters used in the simulation and real application and summarize the results.

*example folder*:

**seed.R:** Generate two parameters used in the real data application

1. $\Tilda{\gamma}$ specified in the estimation of principal stratum probabilities
2. Random seed

**combine_copern.R:** Read in the outputs from each parameter configuration and combine them into a summary table.

*simulations folder:*

**number.R:** Generate four parameters used in the simulation studies

1. $\lambda_0$
2. true value of $\gamma$ used in generating the datasets
3. $\Tilda{\gamma}$ prespecified when estimating principal stratum probabilities
4. Random seed

**combine.R:** Read in the outputs from each parameter configuration and combine them into a summary dataset.

**simulation_summary.R:** Read in the summary dataset and visualize it in a table

*Appendix codes folder:*

**copula_comprison.R:** To compare the fit of bi-variate Clayton, Frank, and Gumbel copulas to the COPERNICUS data.

**example_copula_est.R:** To obtain the point estimate of the treatment effect using the principal stratum probabilities estimated from the Clayton copula for the COPERNICUS data.

**example_copula_CI.R:** To obtain the bootstrap confidence interval of the treatment effect using the principal stratum probabilities estimated from the Clayton copula for the COPERNICUS data.

**example_copula_summary.R:** Combine the results (point estimates and confidence intervals) and visualize them in a table.
